# Supplementary material for: Molecular Identification and Characterization of Two Groups of Phytoplasma and Candidatus Liberibacter Asiaticus in Single or Mixed Infection of Citrus maxima on Hainan Island of China
Source: Biology (Basel). 2022 Jun 6;11(6):869. doi: 10.3390/biology11060869 (PMC9220215; doi:10.3390/biology11060869)
Supplement: Supplementary file 1 [file biology-11-00869-s001.zip › biology-1720014-Supplementary File S1.pdf]

Supplementary gene sequences

16S rRNA gene sequence fragments of the phytoplasma strains CmPII-hn and CmPXXXII-hn, 16S rRNA and β-operon gene sequence fragments of the *Candidatus* Liberibacter asiaticus strain CmLas-hn as well as their GenBank accession numbers are shown as follows:

>CmPII-hn, 16S rRNA gene, partial cds (ON159857)
AGCAACCTACCCTAAAGACGAGGATAACCATTGGAAACAGTGGCTAAGACTGGATAGGAAGAT
AAAAGGCATCTTGTATCTTTTAAAAGACCTAGTTAATAGGTATGCTTTAGGAGGGGCTTGCGCCA
TATTAGTTAGTTGGTAGGGTAATGGCCTACCAAGACGATGATGTGTAGCTGGACTGAGAGGTTG
AACAGCCACATTGGGACTGAGACACGGCCCAAACTCCTACGGGAGGCAGCAGTAGGGAATTTTC
GGCAATGGAGGAAACTCTGACCGAGCAACGCCGCGTGAATGACGAAGTACTTCGGTATGTAAA
GTTCTTTTATCGAGGAAGAAAAGCAAATGGCGAACCATTTGTTTGCCGGTACTTGATGAATAAG
CCCCGGCTAATTATGTGCCAGCAGCCGCGGTAAGACATAAGGGGCAAGTGTTATCCGGAATTAT
TGGGCGTAAAGGGTGCGTAGGCGGTCTAGTAAGTCAGTGGTGTAATGGCAACGCTTAACGTTGT
CCGGCTATTGAAACTGCTAAACTTGAGTTAGATAGAGGCGAGTGGAATTCCATGTGTAGCGGTA
AAATGCGTAAATATATGGAGGAACACCAGAGGCGTAGGCGGCTCGCTGGGTCTTAACTGACGCT
GAGGCACGAAAGCGTGGGGAGCAAACAGGATTAGATACCCTGGTAGTCCACGCCGTAAACGAT
GAGTACTAAGTGTCGGGTTAAACCGGTACTGAAGTTAACACATTAAGTACTCCGCCTGAGTAGT
ACGTACGCAAGTATGAAACTTAAAGGAATTGACGGGACTCCGCACAAGCGGTGGATCATGTTGT
TTAATTCGAAGATACCCGAAAAACCTTACCAGGTCTTGACATGTTTTTGCAAAATGATAGTAATA
TCGTGGAGGTTACCAGAAACACAGGTGGTGCATGGTTGTCGTCAGCTCGTGTCGTGAGATGTTA
GGTTAAGTCCTAAAACGAGCGAAACCCTTATCGTTAGTTGCCAGCACGTTATGGTGGGGACTTTA
ACGAGACTGCCAATGATAAATTGGAGGAAGGTGAGGATCACGTCAAATCAGCATGCCCCTTATG
ACCTGGGCTACAAACGTGATACAATGGCTGTTACAAAGGGTAGCTGAAACGCAAGTTCTTGGCC
AATCCCCAAAAACAGTCCCAGTCCGGATTGAAGTCTGCAACTCGACTTCATGAAGTTGGAATCG
CTAGTAATCGCGAATCAGCATGTCGCGGTGAATACGTTCTCGGGGTTTGTACACACCGCCCGTCA
AACCACGAAAGTTGGCAATACCCCAAAACGGTAGC

>CmPXXXII-hn, 16S rRNA gene, partial cds (ON159856)
CTGCCTTTAAGACGAGGATAACAAGTGGAAACATTTGCTAAGACTGGATAGGAAATAGAAGGAT
AACCTTTTATTTTTAAAAGACCTTCTTCGGAAGGTATGCTTAAAGAAGGGCTTGCGCCACATTAG
TTAGTTGGTAGGGTAAAAGCCTACCAAGACTATGATGTGTAGCTGGACTGAGAGGTTGAACAGC
CACATTGGGACTGAGACACGGCCCAAACTCCTACGGGAGGCAGCAGTAGGGAATTTTCGGCAAT
GGAGGAAACTCTGACCGAGCAACGCCGCGTGAACGAAGAAGTATTTAGGTATGTAAAGTTCTTT
TATTGAAGAAGAAAAAATAGTGGAAAAACTATCTTGACGCTATTCAATGAATAAGCCCCGGCTA
ACTATGTGCCAGCAGCCGCGGTAAGACATAGGGGGCGAGCGTTATCCGGAATTATTGGGCGTAA
AGGGTGCGTAGGCGGTTAGATAAGTCTATAATTTAATTTCAGTGCTTAACGCTGTTCTGTTATAG
AAACTGTCTAGCTAGAGTGAGATAGAGGCAAGTGGAATTCCATGTGTAGCGGTAAAATGTGTAA
ATATATGGAGGAACACCAGAAGCGTAGGCGGCTTGCTGGGTCTTTACTGACGCTGAGGCACGAA

AGCGTGGGTAGCAAACAGGATTAGATACCCTGGTAGTCCACGCCGTAAACGATGAGTACTAAGT
GTCGGGGTTAAACTCGGTACTGAAGTTAACACATTAAGTACTCCGCCTGAGTAGTACGTACGCA
AGTATGAAACTTAAAGGAATTGACGGGACTCCGCACAAGCGGTGGATCATGTTGTTTAATTCGA
AGATACACGAAAAACCTTACCAGGTCTTGACATACTCTGCAAAGCTATAGAAATATAGTGGAGG
TTATCAGGGATACAGGTGGTGCATGGTTGTCGTCAGCTCGTGTCGTGAGATGTTAGGTTAAGTCC
TAAAACGAGCGCAACCCTTGTCGTTAATTGCCAGCACGTTATGGTGGGACTTTAGCGAGACTG
CCAATTAAAAATTGGAGGAAGGTGAGGATTACGTCAAATCATCATGCCCCTTATGATCTGGGCT
ACAAACGTGATACAATGGCTGTTACAAAGAGTAGCTGAAATGCGAGTTTTTAGCCAATCTCAAA
AAAGCAGTCTCAGTTCGGATTGAAGTCTGCAACTCGACTTCATGAAGTTGGAATCGCTAGTAATC
GCGAATCAGCATGTCGCGGTGAATACGTTCTCGGGGTTTGTACACACCGCCCGTCAAACCACGA
AAGTTGATAATAC

>CmLas-hn, 16S rRNA gene, partial cds (ON080846)
AACGTGTGCTAATACCGTATACGCCCTATTGGGGGAAAGATTTTATTGGAGAGAGATGAGCCTG
CGTTGGATTAGCTAGTTGGTAGGGTAAGAGCCTACCAAGGCTACGATCTATAGCTGGTCTGAGA
GGACGATCAGCCACACTGGGACTGAGACACGGCCCAGACTCCTACGGGAGGCAGCAGTGGGGA
ATATTGGACAATGGGGGCAACCCTGATCCAGCCATGCCGCGTGAGTGAAGAAGGCCTTAGGGTT
GTAAAGCTCTTTCGCCGGAGAAGATAATGACGGTATTCGGAGAAGAAGCCCCGGCTAACTTCGT
GCCAGCAGCCGCGGTAATACGAAGGGGGCGAGCGTTGTTCGGAATAACTGGGCGTAAAGGGCG
CGTAGGCGGGCGATTAAGTTAGAGGTGAAATCCCAGGGCTCAACCTTGGAACTGCCTTTAATAC
TGGTTGTCTAGAGTTTAGGAGAGGTGAGTGGAATTCCGAGTGTAGAGGTGAAATTCGTAGATAT
TCGGAGGAACACCGGTGGCGAAGGCGGCTCACTGGCCTGATACTGACGCTGAGGCGCGAAAGC
GTGGGGAGCAAACAGGATTAGATACCCTGGTAGTCCACGCCGTAAACGATGAGTGCTAGCTGTT
GGGTGGTTTACCATTCAGTGGCGCAGCTAACGCATTAAGCACTCCGCCTGGGGAGTACGGTCGC
AAGATTAAAACTCAAAGGAATTGACGGGGGCCCGCACAAGCGGTGGAGCATGTGGTTTAATTCG
ATGCAACGCGCAGAACCTTACCAGCCCTTGACATGTATAGGACGATATCAGAGATGGTATTTTC
TTTTCGGAGACCTTTACACAGGTGCTGCATGGCTGTCGTCAGCTCGTGTCGTGAGATGTTGGGTT
AAGTCCCGCAACGAGCGCAACCCCTGCCTCTAGTTGCCATCAAGTTTAGGTTTTTACCTAGATGT
TGGGTACTTTATAGGGACTGCCGGTGATAAGCCGGAGGAAGGTGGGGATGA

>CmLas-hn, β-operon gene fragment, partial cds (ON098932)
GGATTCCTTTTTCGCTATCGGATCGCTTCTTTTTTGTAAGGGATGCGTTAGGATTTTTGTTCTTCTT
CGAAATCAAGATATGAAAATATTTTCTTGGTATAGATATAGGAAAAGGAATGGGTATATTTGTC
ATCTGGAGATGAAAGTTGAATAGACAAGGAAAGAGCGTAGAAATTTCTGAATTAAGTAAGATTT
TTTCTTCTTCTGGATCAATTGTTGTTGCACATTATAAGGGAATTAGTGTTGCGCAAATTAAAGAT
CTTCGGAAAAAGATGCGGGAAGCTGGTGGAGGTGTAAAAGTTGCCAAAAATCGTCTCGTCAAGA
TTGCTATCCGTGATACTAGTATTAGAGGAATATCTGATCTTTTCGTTGGGCAGTCTCTAATTGTCT
ATTCGGATAGTCCTGTTATTGCTCCTAAAATTTCGGTTAGCTTTTCAAATGACAATAATGAATTTA
GAGTTCTTGGTGGGGTTGTAGAGAAGGGCGTCCTTAATCAAGATTCTATCAAGCAAATTGCTTCT
TTACCCGATCTTGAGGGTATTCGAGCTGGTATCATAAGTGCTATCCAATCTAATGCAACT
